# Antihypertensive Effects of *Artemisia scoparia* Waldst in Spontaneously Hypertensive Rats and Identification of Angiotensin I Converting Enzyme Inhibitors

**DOI:** 10.3390/molecules201119657

**Published:** 2015-11-03

**Authors:** Jeong-Yong Cho, Kyung-Hee Park, Do Young Hwang, Saoraya Chanmuang, Lily Jaiswal, Yang-Kyun Park, Sun-Young Park, So-Young Kim, Haeng-Ran Kim, Jae-Hak Moon, Kyung-Sik Ham

**Affiliations:** 1 Department of Food Biotechnology and Solar Salt Research Center, Mokpo National University, Jeonnam 534-729, Korea; jyongcho@mokpo.ac.kr (J.-Y.C.); adle1004@hanmail.net (K.-H.P.); douyoung533@naver.com (D.Y.H.); bum14b@gmail.com (S.C.); bluelily_bird@yahoo.co.in (L.J.); ykpark@mokpo.ac.kr (Y.-K.P.); sw-chun@hanmail.net (S.-Y.P.); 2 Division of Functional Food & Nutrition, National Academy of Agricultural Sciences, Rural Development Administration (RDA), Jeonbuk 560-500, Korea; foodksy@korea.kr (S.-Y.K.); kimhrr@korea.kr (H.-R.K.); 3 Department of Food Science & Technology, and Functional Food Research Center, Chonnam National University, Gwangju 500-757, Korea; nutrmoon@jnu.ac.kr

**Keywords:** *Artemisia scoparia*, halophyte, blood pressure, ACE inhibitor, phenolics

## Abstract

We investigated the antihypertensive effects of *Artemisia scoparia* (AS) in spontaneously hypertensive rats (SHR). The rats were fed diets containing 2% (*w*/*w*) hot water extracts of AS aerial parts for 6 weeks. The AS group had significantly lower systolic and diastolic blood pressure levels than the control group. The AS group also had lower angiotensin I converting enzyme (ACE) activity and angiotensin II content in serum compared to the control group. The AS group showed higher vascular endothelial growth factor and lower ras homolog gene family member A expression levels in kidney compared to the control group. The AS group had significantly lower levels of plasma lipid oxidation and protein carbonyls than the control group. One new and six known compounds were isolated from AS by guided purification. The new compound was determined to be 4′-*O*-β-d-glucopyranoyl (*E*)-4-hydroxy-3-methylbut-2-enyl benzoate, based on its nuclear magnetic resonance and electrospray ionization-mass spectroscopy data.

## 1. Introduction

High blood pressure is a risk factor for stroke, coronary heart disease and renal vascular disease [1,2]. The control of blood pressure has received considerable attention for public health reasons. Many studies have indicated that foods such as fruits and vegetables have blood pressure-lowering abilities [3,4,5]. Therefore, the clinical importance of fruits and vegetables has received considerable attention.

Halophytes grow widely in the saline environments of coastal sand dunes, salt marshes, mud flats, and inland deserts [6]. They are able to thrive under high salt conditions, although their growth patterns are slightly different depending on the saline concentration. Many studies have indicated that several halophytes, including *Salicornia herbacea*, *Cressia cretica*, and *Suaeda fruticosa*, have various biological effects, including antioxidant, anti-inflammation, and anticancer activities [6,7,8,9]. In addition, halophytes accumulate large amounts of essential minerals, amino acids, and phytochemicals, such as phenolic acids and flavonoids [10,11,12]. These plants accumulate various secondary metabolites including antioxidative compounds with multiple biochemical functions such as the maintenance of ion homeostasis and cell protection in response to saline stress [13,14]. Therefore, halophytes are seen as a potentially useful crop and food source. Recently, we have analyzed the various biological activities of 22 halophytes which were grown in the saline environment of South Korea [15]. *Artemisia scoparia* (AS) had higher angiotensin I converting enzyme (ACE) inhibitory activity than other halophytes tested.

AS belongs to the family of Asteraceae, which includes *A. vulgaris* (mugwort) and *A. capillaris*. The AS aerial parts are eaten as a seasoned vegetable and herb in Korea. The AS has various biological effects, including anti-oxidative, antimicrobial, anti-allergic, anti-atherogenic, anticancer, anti-inflammatory, and liver protection activities [16,17,18,19,20,21]. Chemical constituents of AS, such as flavonoids, coumarins, and essential oils, have been investigated [16,17,18,19,22]. However, studies on the antihypertensive effects of AS in animal models have not yet been performed.

Garlic (*Allium sativum*) is widely used as a seasoning and ingredient in cooking around the world and has been reported to show various biological activities, such as anti-hypertension, anti-oxidant, and anticancer properties [23,24,25,26]. In particular, garlic is well known to be a useful food to prevent high blood pressure [27,28]. The addition of garlic to AS was expected to synergistically increase the high blood pressure prevention effects more than AS alone. Therefore, in this study, the water extract powder of garlic and AS mixture (ASG) was prepared and used in the animal study. In addition, understanding the chemical constituents of AS is very important in acquiring basic information on blood pressure-lowering ability and the value of this plant as food.

In the present study, we evaluated the antihypertensive effects of AS and ASG in spontaneously hypertensive rats (SHR), which have been widely used as an animal model for evaluating the antihypertensive effects of natural products or foods [29]. In addition, the isolation and identification of ACE inhibitors from AS aerial parts, using purification guided by an ACE inhibitory assay, were performed.

## 2. Results and Discussion

### 2.1. Preventive Effects of AS on Blood Pressure in SHR

**Figure 1:**
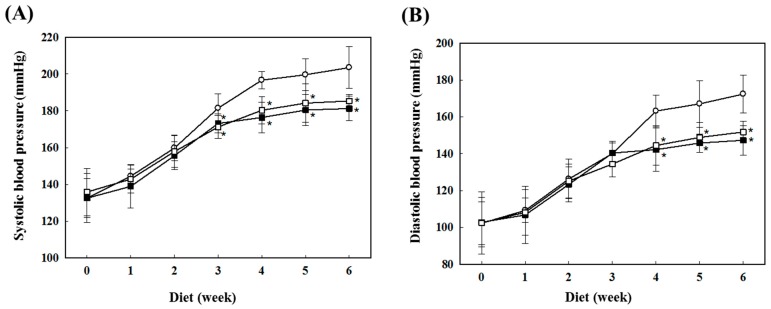
Changes of systolic (**A**) and diastolic (**B**) blood pressure in the rats fed the diets of AS and ASG for 6 weeks. ○, Control; □, AS water extract; ■, ASG water extract. Values are expressed as mean ± SD (*n* = 8). * *p* < 0.05, compared with control (Tukey’s test).

The blood pressure of the SHR fed AS and ASG during 6 weeks was measured using a tail cuff method (Figure 1). The systolic and diastolic blood pressures of the rats fed with normal diet (control) gradually increased during the diet. The AS and ASG diets inhibited the increase of blood pressure in SHR. The blood pressures of the rats fed AS and ASG were similar to that of control rats until 2 weeks into the diet and, thereafter, were significantly lower than that of the control group. At 6 weeks of diet, systolic pressure levels of AS and ASG groups were about 15 mmHg lower than that of the control rats (Figure 1A). The blood pressure of ASG diet group, which was prepared with the addition of garlic to AS, was similar to AS diet group during the diet. This trend in systolic blood pressure was similar to the results of diastolic blood pressure (Figure 1B).

**Figure 2:**
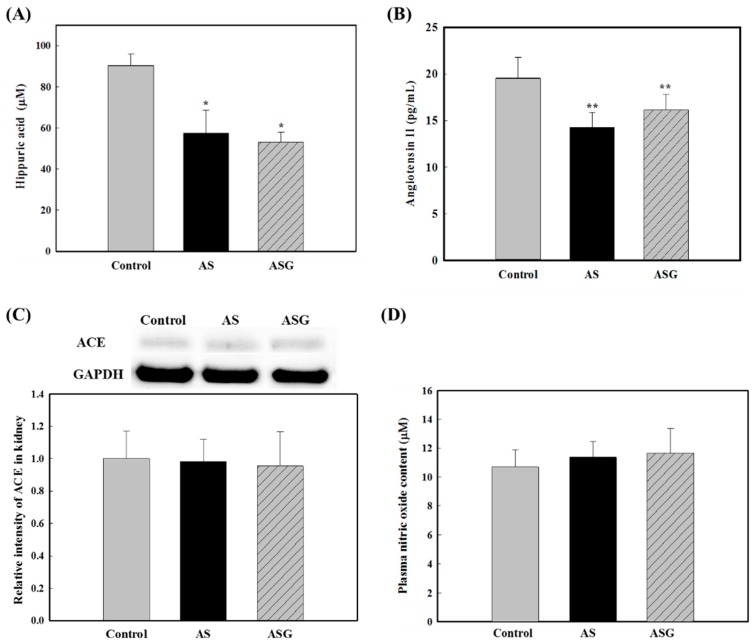
Serum ACE activity (**A**); serum angiotensin II content (**B**); ACE expression level of kidney (**C**); and plasma nitric oxide content (**D**) in rats fed diets of AS and ASG for 6 weeks. Values are expressed as mean ± SD (*n* = 8). * *p* < 0.05 and ** *p* < 0.01, compared with control (Tukey’s test).

The renin-angiotensin-aldosterone system (RAAS) plays an important role in controlling blood pressure in the body [30]. In this system, ACE converts angiotensin I to angiotensin II that increases blood pressure by causing the constriction of blood vessels [31,32]. Angiotensin II also promotes oxidative stress, inflammation and fibrosis [33]. The ACE activities and angiotensin II contents in the blood of the rats fed AS and ASG are shown in Figure 2. The AS and ASG groups exhibited significantly lower ACE activities and angiotensin II contents in the blood than the control rats (Figure 2A,B). However, significant differences in the ACE activities and angiotensin II contents between AS and ASG groups were not observed. These results were consistent with the results of systolic and diastolic blood pressure levels. The reduced ACE activity could be due to the reduced expression of ACE protein or due to the reduced catalytic activity by inhibitors. We analyzed ACE protein expression level in the kidney using immunoblotting. As shown in Figure 2C, there was no significant difference in protein expression levels of ACE among the control, AS, and ASG groups, indicating that the reduced ACE activities of the AS and ASG groups were probably due to the reduced catalytic activity. *In vitro* high ACE-inhibitory activities in AS and ASG, lower ACE activities and angiotensin II contents in the blood of the rats fed AS and ASG, no significant difference in protein expression levels of ACE among the groups, and lower systolic and diastolic blood pressures of the rats fed AS and ASG lead to a possibility that ACE inhibitors in AS and ASG inhibit ACE activity of rats, which results in the reduction of angiotensin II production and blood pressure of the rats fed AS and ASG.

**Figure 3:**
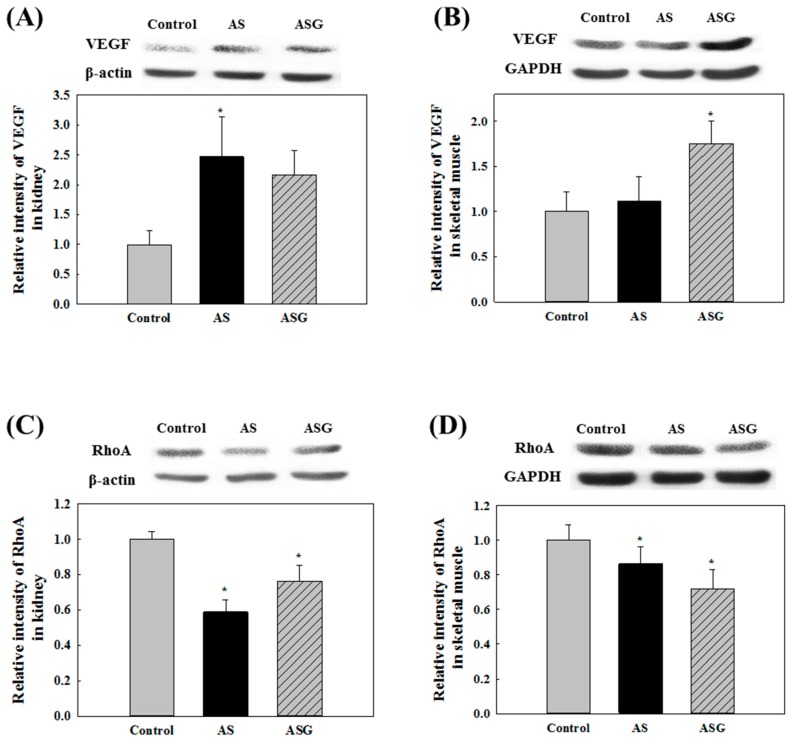
Expression levels of VEGF and RhoA in the rats fed the diet of AS and ASG for 6 weeks. The VEGF expression in kidney (**A**) and skeletal muscle (**B**); The RhoA expression in kidney (**C**) and skeletal muscle (**D**). Values are expressed as mean ± SD (*n* = 8). * *p* < 0.05, compared with control (Tukey’s test).

Endogenous nitric oxide (NO), which is released by endothelial cells of blood vessel through NO synthesis, is a major vasodilator, possibly resulting in lowered blood pressure [34]. No significant difference for the plasma NO concentration among the groups was observed (Figure 2D), although there was a trend that plasma NO concentrations in the rats fed AS and ASG appeared to be slightly higher than that of the control rats. Vascular endothelial growth factor (VEGF) increases NO production by stimulation of endothelial NO synthase (eNOS) and leads to vasodilation [35,36]. In addition, NO suppresses the activation of ras homolog gene family member A (RhoA) that plays a role in regulating vasoconstriction via inhibition of dephosphorylation by myosin phosphatase [37]. Angiotensin II promotes VEGF secretion and the activation of RhoA [38,39]. However, in case of VEGF, relation between VEGF expression and angiotensin II is somehow complicated because ACE inhibition was also reported to induce the expression of VEGF via bradykinin that is increased by ACE inhibition [40,41]. The rats fed AS and ASG had higher levels of VEGF expression and lower levels of RhoA expression in the kidney and skeletal muscle compared to the control rats, although their expression levels were slightly different in each tissue (Figure 3). The AS more efficiently enhanced VEGF expression and suppressed RhoA expression in the kidney. In contrast, the ASG was more effective in the skeletal muscle of SHR. However, there were no statistically significant differences among the AS and ASG groups. These results indicate that AS and ASG reduce blood pressure in SHR via the enhancement of VEGF expression and the suppression of RhoA expression.

In this study, we demonstrated that AS and ASG inhibited ACE activity, but did not increase NO generation. However, no significant difference in NO content and inhibition of ACE activity between AS and ASG was observed, indicating that additional amount of garlic in AS could not synergistically induce an antihypertensive effect. Reduction of blood pressure by AS and ASG could be due to ACE inhibition, decreased oxidative stress, enhanced endothelial function, or direct action on the vascular smooth muscle. Our study demonstrated a possibility that ACE inhibitors in AS played a role in reduction of blood pressure as intake of AS reduced ACE activity and angiotensin II content, but did not suppress ACE expression. There could be another possible mechanism that ACE inhibitors reduce blood pressure. It has been reported that endothelial dysfunction is an early feature of angiotensin II-mediated hypertension [41]. Therefore, it may be possible that reduced angiotensin II content by ACE inhibitors of AS could improve endothelial function and played a role in reduction of blood pressure.

### 2.2. Preventive Effects of AS on Oxidative Stress in SHR

**Figure 4:**
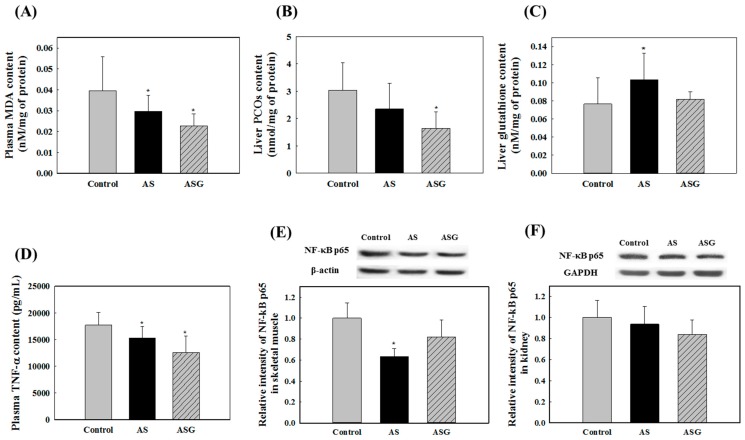
Effects of AS and ASG on oxidative stress in rats. (**A**) Lipid peroxidation in plasma; (**B**) Protein oxidation in liver; (**C**) Glutathione content in liver; (**D**) TNF-α level in plasma; (**E**) NF-κB p65 expression in kidney; (**F**) NF-κB p65 expression in skeletal muscle. Values are expressed as mean ± SD (*n* = 8). * *p* < 0.05, compared with control (Tukey’s test).

Oxidative stress is increased in high blood pressure in response to vessel stimulation [42]. Breakdown of redox balance produces excessive reactive oxygen species and lead to oxidize cellular components such as DNA, RNA, proteins, and lipids. Malondialdehyde (MDA) and protein carbonyls (PCO) are important markers of lipid peroxidation and protein oxidation, respectively [43,44]. In addition, the increase of oxidative stress induces inflammation by activating NF-κB via tumor necrosis factor alpha (TNF-α) [45]. In this study, we found that AS and ASG more efficiently suppressed the increasing levels of oxidative stress- and inflammation-related biomarkers. That is, the rats fed with AS had lower levels of MDA in the plasma and PCO in the liver (Figure 4A,B) than the control rats. In addition, the rats fed with the AS had higher total glutathione content in the liver than the control rats (Figure 4C,D). The rats fed with the AS had lower levels of TNF-α in plasma and NF-κB expression in kidney than the control rats (Figure 4E,F). These results indicate that AS and ASG reduce oxidative stress and inflammation in SHR. Garlic has been reported to inhibit hypertension by suppressing NF-κB expression and lowering ROS concentration [46]. In particular, reduction of oxidative stress and inflammation of ASG was more effective than those of AS, although they were no statistically significant differences between the AS and ASG groups. Therefore, it seemed apparent that reduced oxidative stress by AS and ASG at least partially played a role in lowering of blood pressure.

We found that AS and ASG reduce blood pressure in SHR via the inhibition of plasma ACE activity, the enhancement of VEGF expression, and the suppression of RhoA expression. However, antihypertensive effects between AS and ASG had no significant difference, suggesting that mainly AS has contributed to the antihypertensive effect of ASG. In particular, AS may reduce blood pressure via an inhibition of ACE, one of the important factors in regulation of blood pressure. In addition, ACE inhibitors have been reported to show various preventive effects for heart failure, myocardial infarction, and diabetic nephropathy [37,38,39,40,41,42,43,44,45,46,47,48,49]. Therefore, we attempted the isolation and identification of the ACE inhibitors from AS aerial parts with guided purification of ACE inhibitory assay. The hot water extracts of the AS aerial part were solvent-fractionated to obtain *n*-hexane, chloroform (CHCl_3_), ethyl acetate (EtOAc), water-saturated *n*-butanol (BuOH), and H_2_O fractions. The ACE inhibitory activities of these fractions were evaluated by comparing their activity against ACE extracts of rabbit lung. The CHCl_3_ and EtOAc fractions showed higher ACE inhibitory activity than the other fractions. The *n*-hexane and BuOH fractions showed very low ACE inhibitory activity, similar to the H_2_O fraction. Therefore, we pursued purification of ACE inhibitors from the CHCl_3_ and EtOAc fractions.

**Figure 5:**
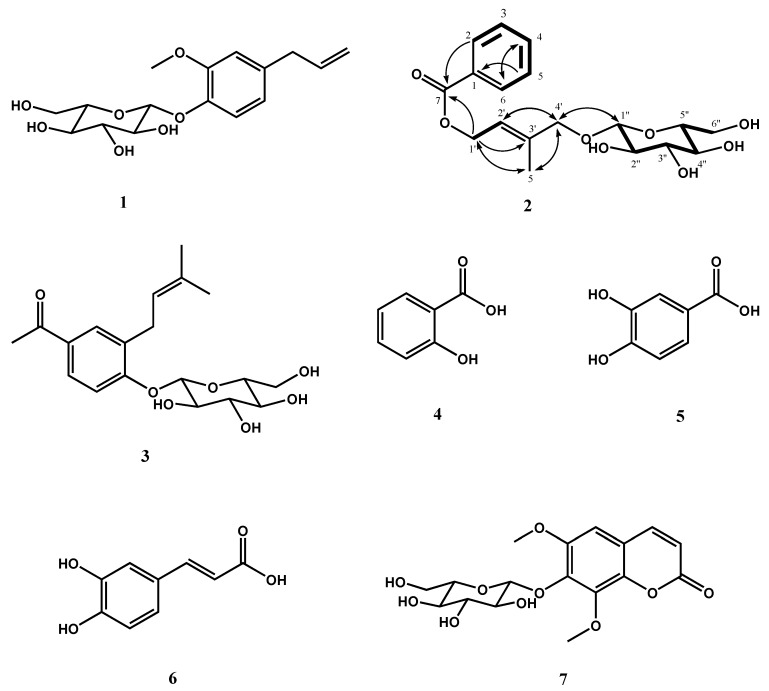
Structures of ACE inhibitors from AS and the important ^1^H-^1^H COSY (bold lines) and HMBC (arrows) correlations for **2**.

Seven ACE inhibitors **1**–**7** were isolated from the CHCl_3_ and EtOAc fractions of AS aerial parts by various column chromatographic procedures with the guided assay of ACE inhibition. The structures of the isolated compounds were determined by NMR and ESI-MS analyses. Among them, six known compounds previously isolated from AS aerial parts were identified as eugenol 2 *O*-β-d-glucopyranoside (**1**), 4-(*O*-β-d-glucopyranoyl)-3-(3′-methyl-2′-butenyl)acetophenone (**3**) [50], salicylic acid (**4**) [51], 3,4-dihydroxybenzoic acid (**5**) [52], caffeic acid (**6**) [52], and isofraxidin 7-*O*-β-d-glucopyranoside (**7**) (Figure 5). Compounds **4**–**6** were identified via comparison of their NMR and MS spectroscopic data with values previously reported in the relevant literature. Compounds **1** and **7** were identified by 1D- and 2D- (HSQC, HMBC, ^1^H-^1^H COSY, and NOE) NMR and MS spectroscopic data. These compounds have been identified from *Michelia hedyosperma* [53] and *Apium graveolens* [54], respectively.

### 2.3. Isolation and Structural Determination of ***2***

The molecular formula of **2** was C_18_H_24_O_8_ (MW, 368), as established by a pseudomolecular ion peak at *m*/*z* 391.1365 [M + Na]^+^ (calculated for C_18_H_24_O_8_Na, *m*/*z* 391.1369, −0.4 mDa) in the HRESI-MS (positive) data (supplementary data). The ^1^H-NMR spectrum exhibited three phenyl group proton signals at δ 8.01 (2H, dd, *J* = 8.4, 1.8 Hz, H-2,6), 7.48 (2H, dd, *J* = 8.4, 8.4 Hz, H-3,5), and 7.60 (1H, tt, *J* = 8.4, 1.8 Hz, H-4) (Table 1). In addition, a *sp*^2^ methine carbon proton signal at δ 5.83 (1H, m, H-2′), two oxygenated *sp*^3^ methylene carbon proton signals at δ 4.31 (1H, d, *J* = 12.6 Hz, H-1′a), 4.11 (1H, d, *J* = 12.6 Hz, H-1′b), and 4.92 (2H, s, H-4′), and one methyl carbon proton signal at δ 1.84 (3H, m, H-5′) were observed in the spectrum, suggesting that the other partial structure was 2-methylbut-2-ene-1,4-diol. The presence of a sugar moiety was derived from the anomeric signal at δ (δ 4.28, H-1′′) and other signals at δ 3.86–3.21 (6H, m, H-2′′–H-6′′). The configuration of β-d-glucopyranoside was assigned by the coupling constant values (*J* = 7.8–9.0 Hz) of sugar proton signals and their proton-proton correlations (Figure 5, bold lines) in the ^1^H-^1^H correlation spectroscopy (COSY) spectrum. This result was also supported by the ^13^C-NMR spectrum, which contained 18 carbon signals, including nine aglycone signals [a carbonyl carbon (δ 168.1, C-7), eight *sp*^2^ carbons (δ 139.6–122.3), two oxygenated *sp*^3^ methylene carbon (δ 62.1, C-1′; 74.6, C-4′) and a methyl carbon (δ 14.5, C-5′)] and six sugar carbon signals (δ 103.3–62.9). The proton signals at 4.92 (2H, s, H-4′) and 3.34 (1H, t, *J* = 9.0 Hz, H-4′′) overlapped with those of the solvents were confirmed in the HSQC spectrum (supplementary data). From the HRESI-MS and 1D-NMR spectra, **2** was suggested to be benzoic acid connected with 2-methylbut-2-ene-1,4-diol and glucose moieties. Complete NMR assignment and connectivity of **2** were further determined by ^1^H-^1^H COSY (Figure 5, bold lines), HSQC, and HMBC (Figure 5, arrows) experiments. In particular, correlation from H-1′ to C-7′ observed in the HMBC spectrum indicated that 2-methylbut-2-ene-1,4-diol was esterified with benzoic acid (Figure 5, arrows). Correlations from H-1′′ to C-4′ and H-4′ to C-1′′ observed in the HMBC spectrum indicated that glucose moiety was etherified with the 4 position of 2-methylbut-2-ene-1,4-diol (Figure 5, arrows). Consequently, the structure of **2** was unambiguously determined to be 4′-*O*-β-d-glucopyranoyl (*E*)-4-hydroxy-3-methylbut-2-enyl benzoate (Figure 5), which is a new compound.

**Table 1:** ^1^H- and ^13^C-NMR data of **2** in methanol-*d*_4_.

Position	2	
	δ_H_ (*rel. int.*, *mult.*, *J* in Hz)	δ_C_
1	-	131.7
2,6	8.01 (2H, dd, 8.4, 1.8)	130.6
3,5	7.48 (2H, dd, 8.4, 8.4)	129.7
4	7.60 (1H, tt, 8.4, 1.8)	134.4
7	-	168.1
1′a	4.31 (1H, d, 12.6)	62.5
1′b	4.11 (1H, d, 12.6)	
2′	5.83 (1H, m)	122.3
3′	-	139.6
4′	4.92 (2H, s) ^(a)^	74.6
5′	1.84 (3H, s)	14.5
1′′	4.28 (1H, d, 7.8)	103.3
2′′	3.21 (1H, dd, 9.0, 7.8)	75.2
3′′	3.34 (1H, t, 9.0) ^(b)^	78.2
4′′	3.28 (1H, t, 9.0)	71.8
5′′	3.24 (1H, m)	78.2
6′′a	3.65 (1H, dd, 12.0, 5.4)	62.9
6′′b	3.86 (1H, dd, 12.0, 2.4)	

^(a), (b)^ The signals of H-4′ and H-3′′ overlapped with those of solvent.

### 2.4. ACE Inhibitory Activity of the Isolated Compounds

**Figure 6:**
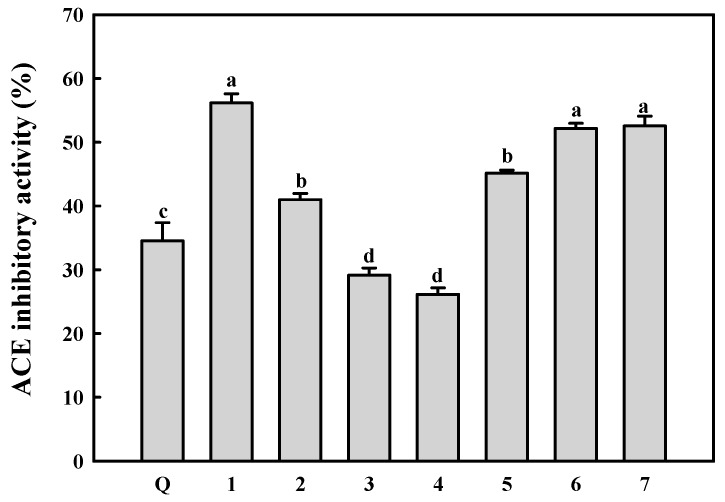
ACE inhibitory activities of ACE inhibitors isolated (**1**–**7**). Q (quercetin) was used as positive control. The isolated compounds were assayed at the concentration of 1 mM. Values are expressed as mean ± SD (*n* = 3). ^a–d^ Results with a different letter differ significantly (*p* < 0.05).

The ACE inhibitory activities of the identified compounds **1**–**7** at the same concentration (1 mM) were evaluated using ACE crude extract from rabbit lung. Quercetin (Q), which is widely distributed in nature and has ACE inhibitory activity [55,56], was used as a positive control. The ACE inhibitory activities of the identified compounds **1**–**7** are shown in Figure 6. As expected, these compounds exhibited ACE inhibitory activities (28.3% ± 1.46%–55.8% ± 1.54%), although their activities were very low in comparison to that (49.3% ± 2.6%) of captopril (1 nM), which is used as a drug. However, the ACE inhibitory activities of the isolated compounds **1**–**7** were higher and similar when compared to that of Q. Compounds **1**, **6**, and **7** had higher ACE inhibitory activity than other compounds. In addition, the new compound **2** had relatively high ACE inhibitory activity at the same concentration (1 mM) under the reaction conditions used. Several phenolic compounds **5**–**7** identified in this study have been reported to show ACE inhibitory activity [54,56,57,58]. These observations indicated that the phenolic compounds identified have *in vitro* ACE inhibitory activities. In addition, phenolic compounds, which are widely distributed in various plants, have been reported to show anti-oxidative and anti-inflammatory activities [59,60]. Therefore, the phenolic compounds identified in this study may be responsible for anti-oxidative activity of AS.

## 3. Experimental Section

### 3.1. General Experimental Procedures

Nuclear magnetic resonance (NMR) spectra were obtained with ^unity^INOVA 600 and 500 spectrometers (Varian, Walnut Creek, CA, USA). Methanol-*d*_4_ containing tetramethylsilane (TMS) was used as solvent. All mass spectra were acquired on a hybrid ion-trap time-of-flight mass spectrometer (SYNAPT G2, Waters, Cambridge, UK) equipped with an electrospray ionization (ESI) source (ESI-MS). Thin-layer chromatography (TLC) was carried out by using silica gel TLC plates (silica gel 60 F254, 0.25 mm thickness, Merck, Darmstadt, Germany) and developed by using a mixture of CHCl_3_/MeOH = 7:3 (*v*/*v*). The fractions were visualized by UV and 1% cerium IV sulfate ethanol solution spray. Silica gel (Kiesel gel 60, 70–230 mesh; Merck, Darmstadt, Germany) and Sephadex LH-20 (25–100 mesh; GE Healthcare Bio-Sciences AB, Uppsala, Sweden) were used for column chromatographies. The fractions obtained from column chromatographies were purified by high performance liquid chromatography (HPLC, Waters, Milford, MA, USA) using a Spherisorb S5 ODS2 column (10 mm × 250 mm). Flow rate was 3.0 mL/min, and eluents were monitored at 220 nm.

### 3.2. Chemicals

The following chemicals were obtained from Sigma Chemical Co. (St. Louis, MO, USA): thiobarbituric acid (TBA), MDA, NADPH, glutathione reductase, l-gluthathione reduced, hydroxylamine hydrochloride, 5,5′-dithiobis(2-nitrobenzoic acid) (DTNB), guanidine hydrochloride, *N*-hippuryl-l-histidyl-l-leucine, hippuric acid, quercetin (Q), and lung acetone powder of rabbit. Rabbit anti-GAPDH polyclonal antibody and anti-β-actin were purchased from Ab Frontier (Seoul, Korea). Horseradish peroxidase (HRP)-conjugated goat anti-rabbit immunoglobin was obtained from Millipore Co. (Billerica, MA, USA). Antibodies against ACE, VEGF, and RhoA were obtained from Santa Cruz Biotechnology (Santa Cruz, CA, USA). Solvents used for analyses were of HPLC grade. All other chemicals were of reagent grade.

### 3.3. Materials

The aerial parts of AS were collected May, 2013 in Shinan County, South Korea. They were washed with water, immediately steamed for 2 min, and dried at 45 °C for 2 days. The dried samples were stored at −20 °C until used in the experiments. Garlic was collected June, 2013 in Shinan County, South Korea. Voucher samples (AS, MNU AS 20130601; garlic, MNU G 20130602) were deposited in the herbarium of the laboratory. The dried AS aerial parts (4 kg) were extracted with distilled water (40 L) at 121 °C for 30 min. After filtration with No. 2 filter paper (Whatman, Maidenstone, UK), the residues were repeatedly extracted with distilled water (20 L), as described above, and then filtered (No. 2 filter paper) again. The extracted solutions were combined and were freeze-dried to obtain AS water extract powder. Additionally, to obtain the water extract powder of AS and garlic mixture, the dried AS aerial part and fresh garlic (ASG) were mixed in the proportion of 3.6:0.4 (*w*/*w*). Extraction, filtration, and freeze-drying of the ASG mixture were performed by the same extraction procedures as described in the preparation of AS powder.

### 3.4. Animal Studies

Male SHR rats (80 ± 10 g, 4 week-old) were obtained from Central Lab. Animal Ltd. (Seoul, Korea). All rats were housed in room temperature (25 ± 1 °C) with humidity (55% ± 5%) and light cycle (12 h: 6:00 to 18:00). Food and water were available *ad libitum*. All rats were fed the standard rodent diet (Harlan Rodent diet, 2018S) for 3 weeks. All experimental procedures were approved by the Ethics Committee of Mokpo National University (no. MNU IACUC-2014-011). The rats used in animal studies were randomly divided into three groups: control group that was fed AIN-93G diet; AS group that was fed the diet containing 2% (*w*/*w*) AS water extract; ASG group that was fed the diet containing 2% (*w*/*w*) ASG water extract. The AS and ASG diets were prepared from 2% (*w*/*w*) AS and ASG water extracts, respectively, based on manufacture protocol of AIN-93G purified diet for rodents. The rats were fed these experimental diets for 6 weeks. Food and water intake were recorded during the AS and ASG diets.

### 3.5. Blood Pressure Measurement

The systolic and diastolic blood pressure levels of rats during the special diets were measured by tail-cuff method using Heater Scanner LE 5650/6 and Storage Pressure Meter LE 5002 (Panlab, S.L., Barcelona, Spain). The rats were placed on a chamber preheated at 37 °C. A clamp type transducers and cuff were set up on the tails of rats. Systolic and diastolic blood pressures of the rats were measured at 37 °C for 10 min. The rats were trained in an acrylic restrainer every day for one week before measuring blood pressure. Systolic and diastolic blood pressures were measured when rats were still unperturbed in the chamber during the inflation-deflation cycles.

### 3.6. Determination of Plasma ACE Activity

ACE activity in serum was measured by using HHL as a substrate [61]. The serum samples (50 μL) were mixed with 0.1 M sodium borate buffer (pH 8.3, 100 μL) containing 300 mM NaCl and 5 mM HHL (50 μL). The mixture was incubated at 37 °C for 45 min, and then the enzymatic reaction was stopped with the addition of 2 N HCl solution (200 μL). The reaction mixture was separated with ethyl acetate (2.0 mL). The upper layer (ethyl acetate layer, 1.5 mL) was concentrated and dissolved with 1 M NaCl solution (1 mL). The absorbance was measured at 228 nm and the hippuric acid content in the serum was calculated using calibration curve of hippuric acid standard.

### 3.7. Determination of Serum Angiotensin II content

Angiotensin II content in serum was determined by using Rat angiotensin II EIA kit (RayBiotech, Inc., Norcross, GA, USA).

### 3.8. Determination of NO Content in Plasma

The NO content in plasma was measured according to Griess assay [62]. The plasma samples (0.1 mL) were mixed with zinc sulfate (50 μL, final concentration 15 mg/mL) and then centrifuged at 1000× *g* for 10 min. The supernatant (100 μL) was mixed with vanadium (III) chloride (100 μL, final concentration 8 mg/mL) and added to 2% sulfanilamide in 5% H_3_PO_4_ solution (50 μL) and 0.1% *N*-(1-naphthyl)ethylenediamine (50 μL). After incubation at 37 °C for 30 min, the absorbance was measured at 540 nm. The NO content in the plasma was calculated using standard curve of sodium nitrite.

### 3.9. Western Blotting

The tissues (0.2 g) were mixed with a lysis buffer containing 1% Triton X-100, 50 mM HEPES (pH 7.4), 100 mM sodium pyrophosphate, 10 mM NF, 10 mM EDTA, 2 mM phenylmethylsulfonyl fluoride, pepstatin (1 μg/mL), leupeptin (1 μg/mL), 10 mM Na_3_VO_4_, and aprotinin (0.1 mg/mL). After protein extraction at 4 °C for 2 h, the homogenate was centrifuged (15,000*× g*, 4 °C) for 40 min. The protein content in the supernatant was determined by using Bradford protein assay [63]. The protein solution (0.1 mL) combined with 0.1 mL of tricine buffer (BioRad, Hercules, CA, USA) was denatured for 5 min at 95 °C. The denatured proteins were analyzed by sodium dodecyl sulfate polyacrylamide gel electrophoresis. The proteins were transferred electrophoretically to polyvinylidene fluoride membranes (Pall Corporation, Pensacola, FL, USA). After blocked with a blocking buffer containing 3% bovine serum albumin (BSA) for 1 h, the membrane was incubated with primary antibodies (VEGF, RhoA, NF-κB p65) overnight at room temperature. After washing, the membrane was incubated with HRP-conjugated goat anti-rabbit immunoglobin G for 1 h and then the bands were visualized by chemiluminescence (ECL) reagent. Visualized protein bands were photographed and their intensities were analyzed by ImageJ software (NIH, Bethesda, MD, USA).

### 3.10. Determination of Thiobarbituric Acid Reactive Substances (TBARS)

The plasma (0.1 mL) was mixed with 0.6 M HCl solution (0.5 mL) containing 20% TCA and 1 M NaOH solution (0.3 mL) containing 0.67% TBA. The mixture was boiled for 20 min, cooled, and then separated with water-saturated *n*-butanol (800 μL). The absorbance of the upper layer (*n*-butanol layer) was measured at 532 nm and TBARS levels in the plasma were calculated using standard curve of MDA [64].

### 3.11. Determination of Protein Carbonyl Content

The protein extract (2.0 mg) obtained from liver tissue was mixed with 0.1 mL of 2.0 M HCl solution containing 10 mM DNPH. The mixture was incubated for 1 h in a dark place at room temperature and heavily shaken at 15 min intervals. The reaction mixture was added to 1.0 mL of a cooled 10% (*w*/*v*) trichloroacetic acid solution and centrifuged at 3000× *g* for 10 min. The protein pellet was washed three times with 2.0 mL of ethanol/ethyl acetate = 1:1 (*v*/*v*). The pellets were then dissolved in 1.5 mL of 6 M guanidine hydrochloride (pH 2.3). After incubation for 10 min at 37 °C, the absorbance was measured at 370 nm. The PCO content was calculated in nmol of carbonyls groups per mg of protein, using the extinction coefficient of 2.2 × 10^4^/M·mL [65].

### 3.12. Measurement of Glutathione (GSH) Content

The liver tissue (0.1 g) was homogenized in 0.05 sodium phosphate buffer (1 mL, pH 7.4) containing 0.14 M KCl and 0.02 M EDTA. The homogenate was mixed with 10% TCA solution (1 mL) and then centrifuged (8000× *g*, 10 min) at 4 °C. The supernatant (0.1 mL) was mixed with 0.1 mL of 0.1 M potassium phosphate buffer containing 5 mM EDTA disodium salt (pH 7.5), 1.68 mM 5,5′-dithiobisnitrobenzoic acid (0.06 mL), glutathione reductase (0.06 mL, 3.33 U/mL), and 0.94 M β-NADPH (0.06 mL). After incubation for 2 min, the absorbance was measured at 412 nm by a spectrophotometer. The GSH content in the liver tissue was determined by a calibration curve prepared with GSH, as a standard [66].

### 3.13. Determination of TNF-α Level in Plasma

The levels of TNF-α in the plasma was measured using an ELISA kit (Koma Biotech Inc., Seoul, Korea) according to manufacturer’s instructions. Briefly, the plasma (0.1 mL) was added in an ELISA kit and incubated for 2 h. After washing with phosphate saline buffer containing 0.0005% Tween-20 (PBST, 4 times), the plate was added to anti-Rat TNF-α (100 μL), incubated for 4 h, and washed with PBST (4 times). The plate was incubated with the addition of streptavidin-HRP conjugate (100 μL) for 30 min followed by treatment of TMB substrate solution (100 μL) for 10 min. After stopping the reaction with 2 M H_2_SO_4_ solution (100 μL), the absorbance of each plate well was measured at 450 nm by ELISA reader (μQuant, Bio-Tek Instruments, Inc., Winooski, VT, USA). The levels of TNF-α in the plasma were quantified from a calibration curve of the standard (47–3000 pg/mL).

### 3.14. Purification and Isolation of ACE Inhibitor in AS Water Extract

The hot water extracts of the dried AS aerial part (1.6 kg dry wt) were suspended in 3.0 L of H_2_O and successively partitioned with *n*-hexane (32 L, three times), CHCl_3_ (32 L, three times), EtOAc (32 L, three times), and BuOH (32 L, three times). Each layer was evaporated *in vacuo* at 38 °C. The combined CHCl_3_ and EtOAc layer (36.4 g) were fractionated on a silica gel column (800 g, 6.0 mm × 30 cm) and eluted with solvent mixtures of CHCl_3_/MeOH (9:1, 7:3, 5:5, 3:7, 0:10, *v*/*v*, each 8 L) to give 11 active fractions (A–K). Fraction C (CHCl_3_/MeOH = 9:1, *v*/*v*, 2.1 g) was refractionated by silica gel column chromatography (60 g, 2.5 × 25 cm) eluting with a series of solvent mixtures of CHCl_3_/methanol (95:5 and 90:10, *v*/*v*, each 600 mL) to give 11 subfractions C1–C11. Subfraction C10 (eluted with CHCl_3_/MeOH = 90:10, *v*/*v*, 61.1 mg) was subjected to semi-preparative ODS-HPLC using an isocratic system of 20% MeCN to yield fraction C10a (*t_R_* 5.5 min, 8.7 mg), **1** (*t_R_* 8.2 min, 2.3 mg), **2** (*t_R_* 10.5 min, 1.1 mg), **3** (*t_R_* 12.3 min, 2.5 mg), and four unknown compounds (*t_R_* 13.3–18.4 min, 8.3 mg).

Fraction D (CHCl_3_/MeOH = 7:3, *v*/*v*, 2.0 g) was refractionated by silica gel column chromatography (40 g, 3.0 × 18 cm) eluting with a series of solvent mixtures of *n*-hexane/EtOAc/methanol (4:8:1, 2:10:1, and 0:12:1, *v*/*v*, each 400 mL) to obtain 11 subfractions (D1–D11). Subfraction D3 (*n*-hexane/EtOAc/methanol = 4:8:1, *v*/*v*, 352.7 mg) was purified by Sephadex LH-20 column chromatography (1.5 mm × 85 cm) eluting with 100% MeOH to afford **4** [elution volume/total volume (Ve/Vt) = 1.01–1.08, 22.5 mg], **5** (Ve/Vt = 1.09–1.16, 55.1 mg), and **6** (Ve/Vt = 1.17–1.32, 200.6 mg). Subfraction D9 (*n*-hexane/EtOAc/methanol = 0:12:1, *v*/*v*, 9.5 mg) was subjected to semi-preparative ODS-HPLC using an isocratic system of 15% MeCN to isolate **7** (*t_R_* 11.5 min, 1.2 mg).

### 3.15. 4′-O-β-d-Glucopyranoyl (E)-4-hydroxy-3-methylbut-2-enyl benzoate *(**2**)*

White amorphous powder. ^1^H- and ^13^C-NMR data shown in Table 1; HRESI-MS (positive) *m*/*z* 391.1365 [M + Na]^+^ (calculated for C_18_H_24_O_8_Na, *m*/*z* 391.1369, −0.4 mDa).

### 3.16. Assay for ACE Inhibitory Activity

The ACE inhibitory activities of the fractions and the isolated compounds were measured according to the methods described by Cushman *et al.* [48], with slight modification. The samples (20 μL) were mixed with 0.1 M sodium borate buffer (pH 8.3, 130 μL) containing 300 mM NaCl and 5 mM HHL (50 μL). The mixture was then preincubated at 37 °C for 10 min. After preincubation, the mixture was added to 100 μL of ACE extract from lung acetone powder of rabbit. The mixture was incubated at 37 °C for 90 min and then the enzymatic reaction was stopped by the addition of 2 N HCl solution (200 μL). The reaction mixture was separated with ethyl acetate (2 mL). The upper layer (ethyl acetate layer, 1.5 mL) was concentrated and the hippuric acid fraction was dissolved with 1 M NaCl solution (1 mL). The absorbance (A) was measured at 228 nm. Q was used as a positive control. The absorbance (B) of Q and the samples was measured by the same procedure as described above except for ACE extract. The ACE inhibitory activities of the samples were determined as the percentage decrease in absorbance compared to that of a control test. The dose-response curve of each compound at the concentration of ≤1 mM was a linear for ACE inhibitory activity. The isolated compounds were assayed at the concentration of 1 mM.


ACE inhibitory activity (%) = [(control − (A − B)/control] × 100

### 3.17. Statistical Analysis

Data are expressed as mean ± standard deviation (SD) using the SPSS, (IBM, Armonk, NY, USA) 19.0 package program. Statistical differences were measured by one-way analysis of variance followed by Tukey’s multiple test. A *p* < 0.05 was considered significant.

## 4. Conclusions

AS attenuated an increment of blood pressure in SHR via the enhancement of VEGF expression and the suppression of RhoA expression by reduced ACE activity and angiotensin II production. However, in this study, we could not see significant synergistic effects of garlic with AS in lowering blood pressure. Possible explanations are as follows: (1) not enough garlic was added to AS; (2) sulfur compounds having antihypertensive activity are volatile and lipophilic. They might not be extracted when garlic and AS were extracted with water in autoclave; (3) garlic may not be synergistic with AS although we do not understand the mechanism. Further studies are required. Reduced ACE activity and angiotensin II content in the plasma of the rats fed AS must have been probably or at least partially due to the ACE inhibitors present in AS. These results led to isolation and identification of ACE inhibitors present in AS for the utilization of this plant as a food and medicinal herb. Seven ACE inhibitors, including one new phenolic glycoside, were isolated and identified from the aerial parts of AS with guided purification of ACE inhibitory assay. Further studies on how much these ACE inhibitors contribute to the antihypertensive effects of AS and how they are absorbed in the body remain to be investigated.

## Supplementary Materials

Supplementary materials can be accessed at: http://www.mdpi.com/1420-3049/20/11/19657/s1.
