# Supplementary material for: Antihypertensive Effects of Artemisia scoparia Waldst in Spontaneously Hypertensive Rats and Identification of Angiotensin I Converting Enzyme Inhibitors
Source: Molecules. 2015 Nov 3;20(11):19789–804. doi: 10.3390/molecules201119657 (PMC6332079; doi:10.3390/molecules201119657)
Supplement: Supplementary file 1 [file molecules-20-19657-s001.pdf]

molecules

Supplementary Informations

NMR and MS Spectra of 2

^{1}H-NMR Spectrum (500 MHz, CD3OD)

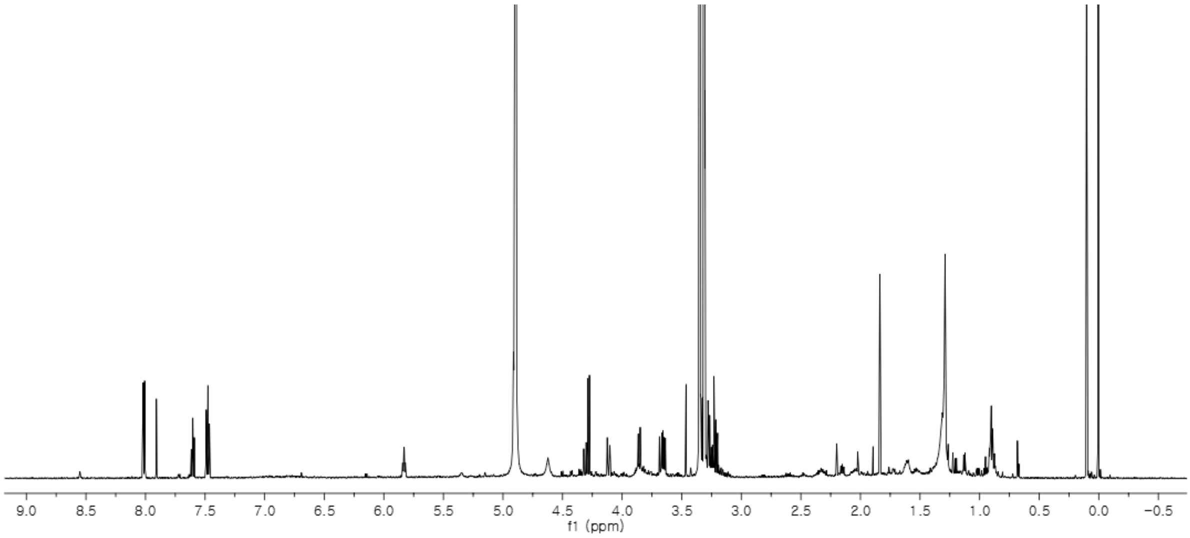

^{13}C-NMR Spectrum (125 MHz, CD3OD)

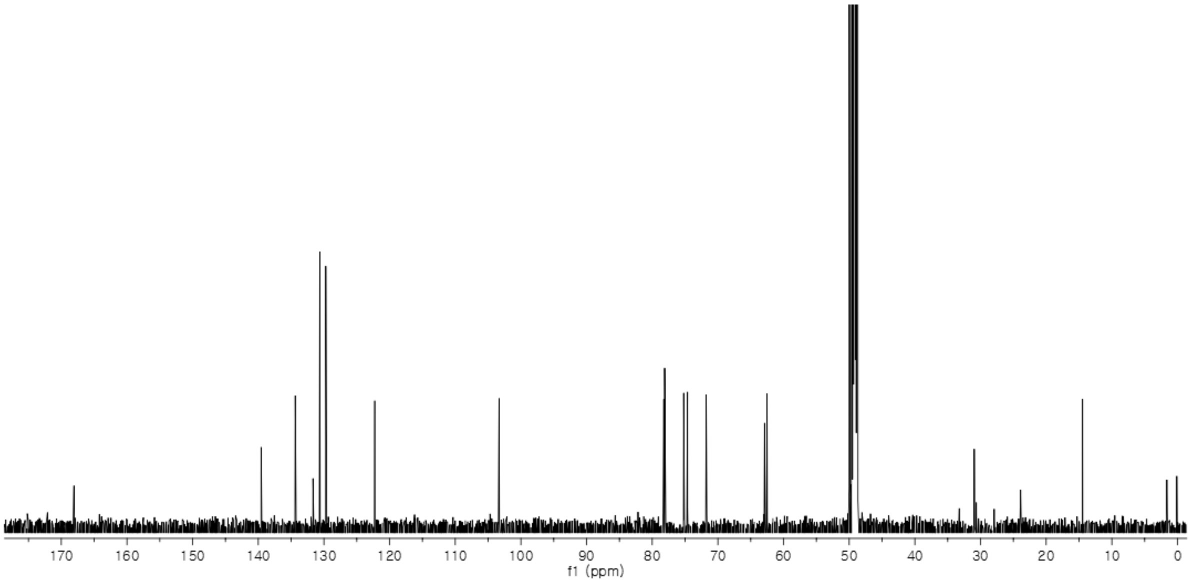

^1H-^1H COSY Spectrum

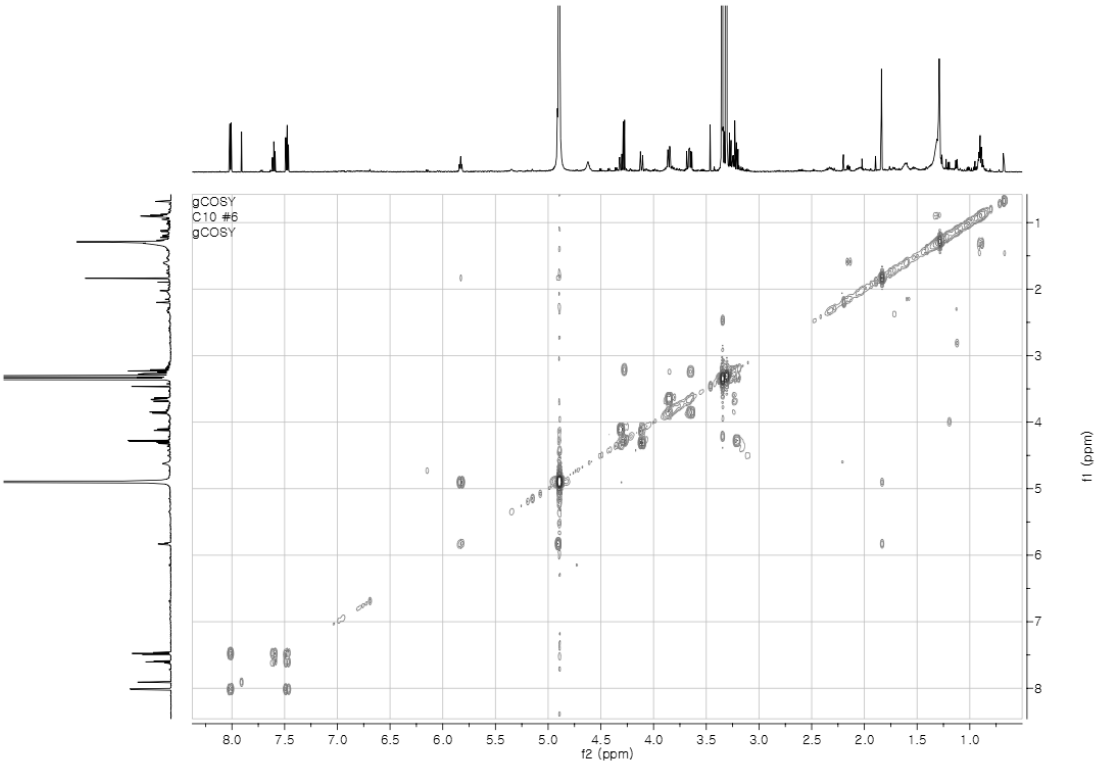

HSQC Spectrum

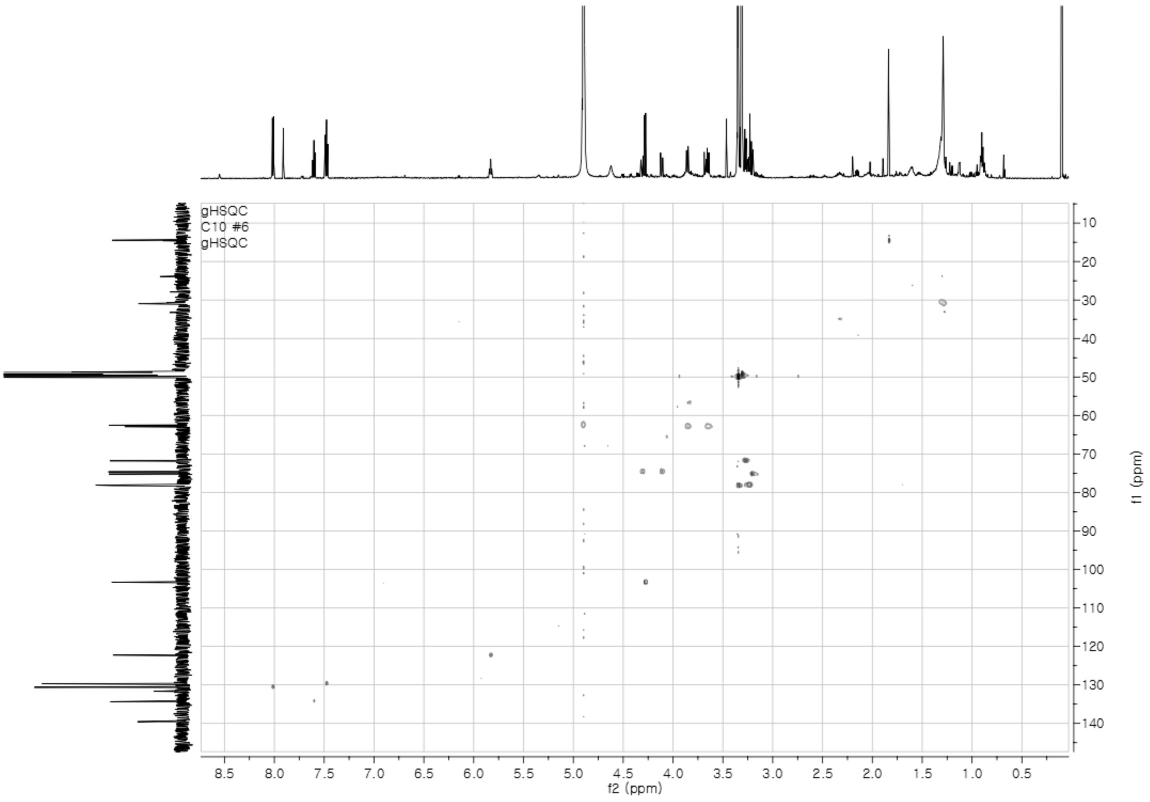

HMBC Spectrum

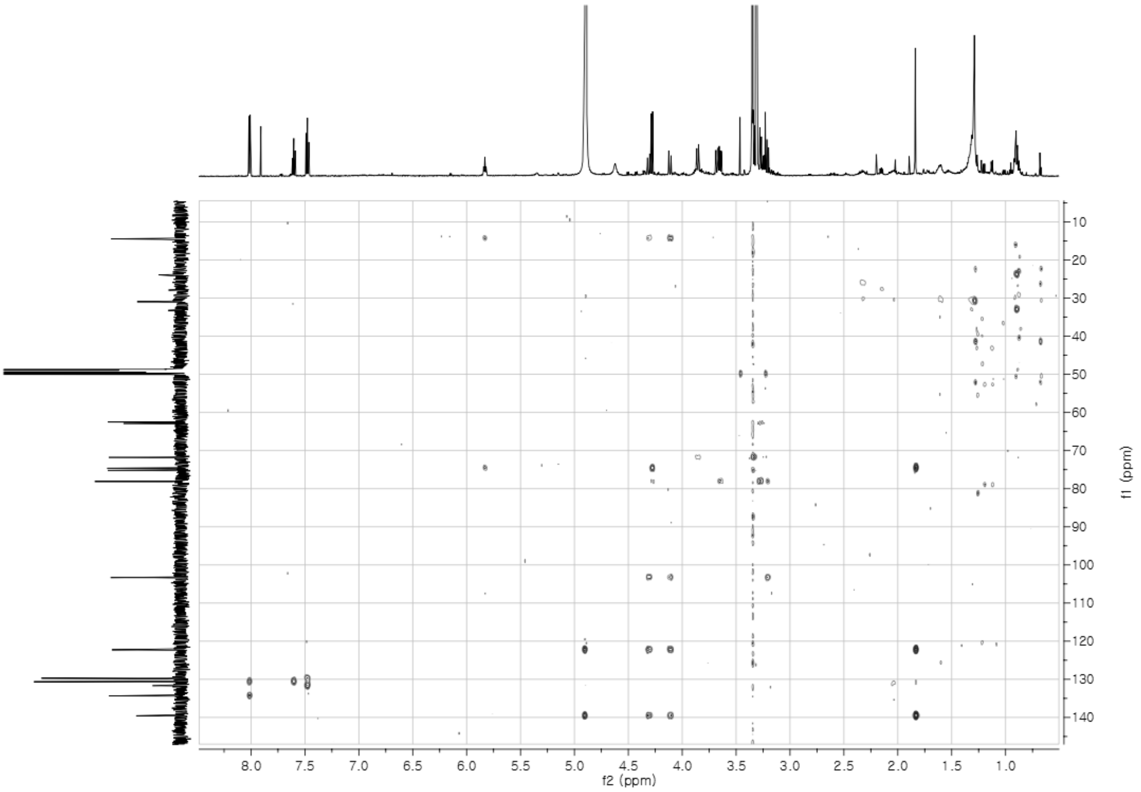

ESI-MS (Positive) Spectrum

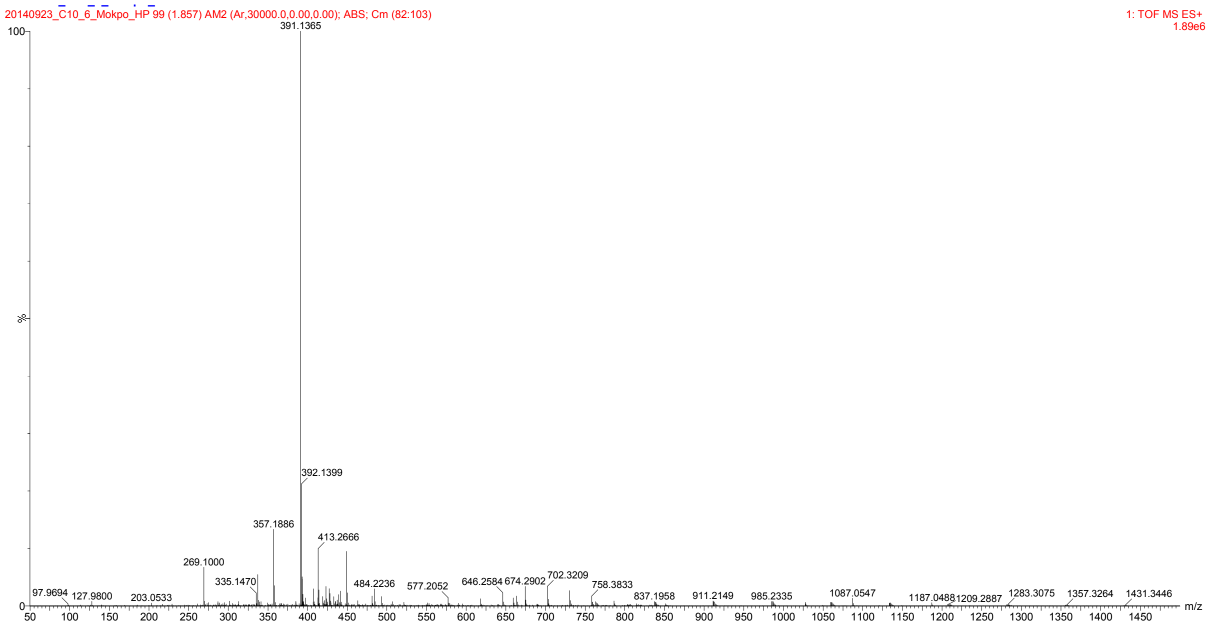

Elemental Composition Report

Single Mass Analysis
Tolerance = 5.0 PPM / DBE: min = −1.5, max = 50.0
Element prediction: Off
Number of isotope peaks used for i-FIT = 3
Monoisotopic Mass, Even Electron Ions
13 formula(e) evaluated with 1 results within limits (all results (up to 1000) for each mass)
Elements Used:

Elements Used: C: 1-20 H: 1-30 O: 1-10 Na: 1-1

Minimum				-1.5				
Maximum		5.0	5.0	50.0				
Mass	Calc. Mass	mDa	PPM	DBE	i-FIT	Norm	Conf(%)	Formula
391.1365	391.1369	-0.4	-1.0	6.5	1078.1	n/a	n/a	C18 H24 O8 Na
